# Electron spin resonance in microalgae whole-cells to monitor hydrogen production

**DOI:** 10.1007/s00775-025-02113-0

**Published:** 2025-03-24

**Authors:** Silvia Pizzanelli, Emanuela Pitzalis, Simone Botticelli, Fabrizio Machetti, Cecilia Faraloni, Giovanni La Penna

**Affiliations:** 1 Institute of Chemistry of Organometallic Compounds, National Research Council, via Moruzzi 1, 56124 Pisa, Italy; 2 Department of Physics, University of Roma Tor Vergata, via della Ricerca Scientifica 1, 00133 Rome, Italy; 3 Section of Roma Tor Vergata, National Institute for Nuclear Physics, via della Ricerca Scientifica 1, 00133 Rome, Italy; 4 Institute of Bioeconomy, National Research Council, via Madonna del Piano 10, 50019 Sesto Fiorentino, Florence, Italy; 5 Institute of Chemistry of Organometallic Compounds, National Research Council, via Madonna del Piano 10, 50019 Sesto Fiorentino, Florence, Italy; 6 Institute of Chemistry of Organometallic Compounds c/o University of Florence, Department of Chemistry “Ugo Schiff”, National Research Council, via della Lastruccia 13, 50019 Sesto Fiorentino, Florence, Italy

**Keywords:** Electron spin resonance, Biological hydrogen, Hydrogenase, Microalgae

## Abstract

**Abstract:**

Unicellular algae can produce pure hydrogen gas from water and sun-light. We observed *Chlorella vulgaris* whole cells when they produce hydrogen using X-band continuous-wave electron spin resonance (ESR). Whole-cell spectroscopy is particularly useful in those cases where purified enzymes are sensitive to oxidant air conditions. By tuning cell preparation, the microwave power, the temperature, the time of air exposure, we could isolate from the background signal candidate markers of hydrogen production. Our observations indicate the presence of a species consistent mainly with an intermediate ${\hbox {Fe}_{3}\hbox {S}_{4}{^{+}}}$ cluster when hydrogen production is high, but not maximal, and when FeS cluster oxidation has just begun. The optimal conditions to detect the above marker by ESR have been identified. Our investigation paves the way to extensive statistical analysis of cellular conditions in future studies using whole-cell ESR.

**Graphical abstract:**

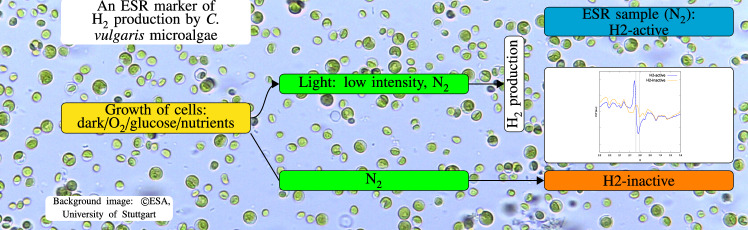

**Supplementary Information:**

The online version contains supplementary material available at 10.1007/s00775-025-02113-0.

## Introduction

Unicellular algae have the property of producing hydrogen as a pure gas under particular conditions, using sun light as energy source and water as proton source [30]:

1 $$\begin{aligned} 2\hbox {H}_{2}\hbox {O} + \hbox {light} \longrightarrow \hbox {O}_{2} + 2\hbox {H}_{2}. \end{aligned}$$

The most studied unicellular alga with this property is *Chlamydomonas reinhardtii* [20, 33] (*Cr*, hereafter) that is efficient in $\hbox {H}_{2}$ production under anaerobiosis, but sensitive to molecular oxygen (dioxygen) [12, 13]. Dioxygen is formed together with hydrogen (see Eq. 1) thus behaving as a negative feedback to reaction. Dioxygen induces the inactivation of the enzyme responsible of hydrogen production (hydrogenase), switching on the usual autotrophic metabolism of algae, made of photosynthesis/respiration cycles. Because of dioxygen sensitivity, hydrogen production lasts only for a few days in cell cultures. Therefore, unicellular algae with dioxygen sensitivity lower than *Cr* have been investigated as potential biological sources of hydrogen for times longer than *Cr*. Alternative algae are of *Chlorella vulgaris* species (*Cvu*, hereafter) for which two promising strains are under intense study: 211/11P [5] and g120 [19, 34].

The *Cvu* cells have the advantage of combining: easy growing conditions; efficient $\hbox {H}_{2}$ production; sensitivity to dioxygen, when producing $\hbox {H}_{2}$, lower than *Cr*. The latter property allows, in particular, the use of $\hbox {N}_{2}$ stream in place of cumbersome manipulations of samples in glove-box, like those required for *Cr* and *E. coli* expressing [FeFe] hydrogenase (see below).

The characterization at molecular level of the peculiar state of *Cvu* cells when the hydrogen production is maximal is missing. The active site of [FeFe] hydrogenase, the enzyme catalyzing the reduction of water to $\hbox {H}_{2}$, contains CO and $\hbox {CN}^{-}$ ligands. Vibrational properties of these ligands have been used to monitor the enzyme state during different phases of cell life [2]. Other markers of hydrogenase state are the magnetic properties of the di-iron center in the same active site, probed by electron spin resonance (ESR) [16]. These properties have been widely investigated in purified hydrogenase [2], when available, and more recently in whole *E. coli* recombinant cells [18, 21]. No crystal structure of *Cvu* [FeFe] hydrogenase is available, thus preventing X-ray diffraction studies. No X-ray absorption studies, similar to those made on *Cr* [32], have been performed yet. Also, no solution NMR studies [28] have been performed on *Cvu*. Only a model of *Cvu* [FeFe] hydrogenase architecture has been recently proposed by computational methods [3]. Genetic engineering of *Cvu* cells is still a research topic [17, 29] also because the biochemical pathways related to hydrogen production are not yet well understood.

Starting from different cell cultures, in this work *Cvu* cells were prepared in two states (see Graphical abstract):

1. H2-inactive state, i.e., a state immediately preceding the $\hbox {H}_{2}$ production, where the control cells display normal replication after heterotrophy at dark and aerobic conditions.
2. H2-active state, i.e., a state where the production of $\hbox {H}_{2}$ starts from the H2-inactive state after weak phototrophy under anaerobiosis;

We used X-band continuous-wave ESR to characterize the two states, tuning the time of light exposure before $\hbox {H}_{2}$ production, the microwave power, the temperature, and the time of exposure to air of the cells after achieving $\hbox {H}_{2}$ production. Our aim is at identifying signals characteristic of the state of *Cvu* cells when $\hbox {H}_{2}$ production is high (H2-active state). In spite of strong overlap among a multitude of signals, as expected in a whole cell, we could identify candidate signals that uniquely mark the H2-active state at the early stages of production. Our analysis supports that the marker is an immature and transient $\hbox {Fe}_{3}\hbox {S}_{4}{^{+}}$ cluster. Finally, changes in distributions of reduced manganese and oxidized high-spin iron were detected and analyzed.

## Methods

### Cell culture

*Cvu* cells were prepared in the H2-inactive state as in the following. Cells from *C. vulgaris* g120 strain were grown in heterotrophy in sterilized (steam autoclaving at 120$^\circ$ C for 30 min) 5 l Erlenmeyer flask, shaken in the darkness in standard growth media (3 l in volume). We used the K3 growth medium in all cell preparations. The composition of the growth medium is reported in Table 1.

**Table 1:** Concentration of nutrients in K3 growth medium

Species	Concentration (g/l)
Glucose	10
Urea	–
$\hbox {KNO}_{3}$	2
$\hbox {KH}_{2}\hbox {PO}_{4}$	0.33
$\hbox {MgSO}_{4}\cdot 7\hbox {H}_{2}\hbox {O}$	0.55
	Concentration (mg/l)
$\hbox {CaCl}_{2}\cdot 2\hbox {H}_{2}\hbox {O}$	174
$\hbox {H}_{3}\hbox {BO}_{3}$	18.5
$\hbox {CuSO}_{4}\cdot 5\hbox {H}_{2}\hbox {O}$	7.44
$\hbox {ZnSO}_{4}\cdot 7\hbox {H}_{2}\hbox {O}$	8.58
$\hbox {CoSO}_{4}\cdot 7\hbox {H}_{2}\hbox {O}$	8.4
$\hbox {MnCl}_{2}\cdot 4\hbox {H}_{2}\hbox {O}$	6.3
($\hbox {NH}_{4})_{6}\hbox {Mo}_{7}\hbox {O}_{24}.4\hbox {H}_{2}\hbox {O}$	6
$\hbox {FeNa(EDTA)}\cdot \hbox {H}_{2}\hbox {O}$	111
$\hbox {Fe}(\hbox {SO}_{4})\cdot 7\hbox {H}_{2}\hbox {O}$	–

Growth was performed in aerobiosis (air at 2% $\hbox {CO}_{2}$) until late-exponential growth rate is achieved at the temperature of 35–37 $^\circ$C (1 week). Cells were collected from the same production flask, put in an Eppendorf, centrifuged and washed with TRIS-HCl three times under $\hbox {N}_{2}$ stream, removing the surnatant each time. $\hbox {N}_{2}$ was bubbled through TRIS–HCl stock solution for several minutes before use. The final precipitate was put in an Eppendorf and finally frozen at $-20\,^\circ$C to store the H2-inactive sample (I1 and I2, hereafter). Each Eppendorf contained approximately 100 $\upmu$l of cell suspension.

H2-active state was reached injecting H2-inactive cells taken from the production flask into a sealed flask exposed to artificial light at 300 $\upmu$mol photons m$^{-2}$ s$^{-1}$, from one side. These conditions are those of weak illumination used to start $\hbox {H}_{2}$ production [19]. After 2 h of weak illumination gas bubbles production is observed. The gas was taken from head-space and analyzed via gas chromatography, confirming the pure $\hbox {H}_{2}$ gas production. Samples of cells were collected from the sealed flask after 2 and 10 h of light irradiation. Each sample was then centrifuged and washed using the same procedure as H2-inactive sample. These samples were indicated as A1 and A2 when, respectively, irradiated for 2 and 10 h. Also in these samples each Eppendorf contained approximately 100 $\upmu$l of cell suspension. In preparation of A1 we also exposed the H2-active sample to air. Samples extracted from the H2-active flask were stirred in a beaker in air for 1 and 30 min, and finally frozen (A1$_0$, A1$_1$, and A1$_{30}$ samples).

For I2 and A2 samples, 9 replicates were collected. Replicates were: 6 for ESR measurements, 3 for samples exposed to CO (see below); 9 for cell counting; 3 for ICP-OES measurements.

The complete list of produced and analyzed samples is summarized at the beginning of Results section.

### ESR sample preparation

I1, A1, I2, and A2 samples collected according to the procedure outlined in Sect. 2.1 were rapidly (1–2 min) unfrozen under $\hbox {N}_{2}$ stream. Then a volume of 250 $\upmu$l of TRIS-HCl was added into the Eppendorf for I1 and A1, to achieve a viscosity suitable to transfer cells into an ESR tube. For I2 and A2 sample, a volume of 500 $\upmu$l of TRIS–HCl was added and about one half of the diluted sample was injected into the ESR tube, while the other half was kept for cell counting. The presented spectra of I2 and A2 samples are the average of 6 replicates. The ESR tubes were filled up to $\sim$4 cm in height. Quartz tubes (707-SQ-250) with an outer diameter of 4 mm and an inner diameter of 3 mm were used. Thus, ESR tubes contained 250–300 $\upmu$l of cell suspension.

To compare $\hbox {Mn}^{2+}$ lineshape in whole cells samples with reference spectra, we prepared two samples, as described in the following. (i) The 1:1 complex of $\hbox {Mn}^{2+}$ with bovine serum albumin (BSA). The MnBSA complex was prepared by adding one equivalent of BSA (Roche Diagnostics Corp.) to a $\hbox {Mn}^{2+}$ solution characterized by a concentration of 0.186 mM. The $\hbox {Mn}^{2+}$ solution was prepared dissolving 1 mg of $\hbox {MnBr}_{2}$ (Fluka 63551) in 25 ml of TRIS–HCl. (ii) The $\hbox {Mn}^{2+}$ aquaion at a concentration of 0.085 mM was prepared by dissolving $\hbox {Mn}(\hbox {NO}_{3})_{2}.4\hbox {H}_{2}\hbox {O}$ (Emsure 20694-39-7) in the same TRIS–HCl buffer used to dissolve cells.

To check if some ESR signals are sensitive to CO binding to metal species, 3 replicates of I3 and 3 replicates of A3 were exposed to a flow of pure CO for 1 h after unfreezing (8 h under $\hbox {N}_{2}$ stream) and before injection into the ESR tube. This exposure was performed replacing the $\hbox {N}_{2}$ stream with CO stream at 1 bar. The procedure was similar to that reported in the literature [6]. Samples are indicated as I2$_\textrm{CO}$ and A2$_\textrm{CO}$.

### Cell counting

The cell density of all samples was measured by cell counts at the microscope. In I1 and A1 samples the count was performed after extracting the cells from the ESR tube after ESR experiments. For these samples, cell integrity was verified. For I2 and A2 samples, the count was performed on the fraction of sample described in Sect. 2.2.

The values of cell density reported in Results are averaged over 9 replicates of I2 and A2 samples. The error is calculated as the standard error.

### ESR experiments

The ESR experiments were performed with two spectrometers. One was a Bruker Elexsys E580 spectrometer provided by the INSTRUCT-ERIC EU infrastructure available at CERM/CIRMMP in Florence (Italy). The second one was a Bruker Elexsys E500 spectrometer provided by the Chemistry Department “Ugo Schiff” (DICUS) of the University of Florence (Italy). X-band (9.4 GHz) continuous-wave (CW) ESR spectra were collected with both instruments using the Elexsys Super High Sensitivity Probehead (ER 4122SHQE) resonator. The temperature was controlled with an Oxford helium temperature regulation unit (4–300 K) in both cases (Oxford Instruments ESR 900). In order to probe saturation of different signals, the microwave power was changed from 0.0005 to 200 mW. The magnetic field modulation amplitude was 10 G; the modulation frequency was 100 kHz; the receiver gain was 60 dB; the number of scans was 1. Most of these parameters were taken from the ESR experiments performed on *Cr* hydrogenase (HydA1) expressed in recombinant *E. coli* engineered cells [21].

The spectra that are shown in this work are the result of the subtraction of the cavity signal. Spectra were all collected at 1024 points of magnetic field. Baseline correction was performed using the first and last 75 points when the field window was 5000 G and first and last 150 points when the window was 1000 G. A spline curve was calculated by interpolating these points and the resulting curve was subtracted to the signal. This processing was performed using programs written in R language after conversion of raw data into text files. An example of base-line correction is displayed in supplementary information (Fig. S1). In order to estimate the relative amount of the ESR species, we used ESR spectra recorded at a microwave power (20 $\upmu$W) below saturation of signals. The ratio *R* between concentration of ions per cell (*c*) in different states is given by the following equation:

2 $$\begin{aligned} R = \frac{c(A)}{c(B)} = \frac{I(A)}{I(B)} \frac{\rho (B)}{\rho (A)}~~. \end{aligned}$$

*A* and *B* indicate the H2-inactive and H2-active state, respectively. As for $\hbox {Mn}^{2+}$ *I* is the intensity of the first peak on the lefthand side of the $\hbox {Mn}^{2+}$ sextet in the derivative spectra. As for $\hbox {Fe}^{3+}$ *I* is the intensity of the maximum of high-spin $\hbox {Fe}^{3+}$ signal. $\rho$ is the the cell density in the respective samples.

### ICP-OES measurements

In these measurements, the final precipitate of I2 and A2 samples, prepared with the procedure described in Sect. 2.1, was analyzed. The whole content of each Eppendorf was treated with 2 ml $\hbox {HNO}_{3}$ (Fluka, TraceSELECT™ for trace analysis >69%) and 1 ml $\hbox {H}_{2}\hbox {O}_{2}$ (Supelco, Suprapur™, 30%) and heated for 2 h at boiling temperature. The mixture was then diluted to 10 ml with ultra-pure water (18.2 M$\Omega$ cm, PureLab Pro, ELGA LabWater, High Wycombe, Bucks, UK) for ICP-OES analysis. After digestion, the solutions of three different replicates of each chosen sample were all clear and homogeneous.

Metals content determination was carried out with an inductively coupled plasma optical emission spectrometer (ICP-OES) Optima 8000 ICP-OES (Perkin Elmer, Waltham, MA, USA) operating at 1500 W and equipped with an autosampler S10, MiraMist™ Nebulizer and cyclonic chamber. Argon (420.069 nm) was used as the internal standard. Calibration solutions were obtained by dilution of commercial standard solutions (Fluka TraceCERT™) in $\hbox {HNO}_{3}$ 2%. Nickel, cobalt, copper, iron and manganese emission signals were measured at 231.604, 228.616, 327.393, 238.204, 257.610 nm, respectively.

For both I2 and A2 samples, 3 replicates were analyzed. The numbers of Fe and Mn atoms per cell reported in Table 3 were derived considering the average values of the metal amount determined by ICP-OES and the cell density (Sect. 2.3).

## Results

In this work, H2-active and H2-inactive samples were investigated using ESR. In order to highlight the effect of the preparation conditions on the ESR spectral features, we investigated samples prepared using two different irradiation times, 2 (preparation 1) and 10 (preparation 2) hours, to reach the H2-active state. A complete list of the studied samples together with their preparation conditions is provided in Table 2. The names of the samples reflect their H2-active (A) or H2-inactive (I) state and the preparation conditions, 1 or 2. In preparation 1, 3 replicates of the A sample were also exposed to air for different amounts of time, which is reflected in the subscripts 0, 1, 30, indicating the minutes of exposure. In preparation 2, 3 replicates of A and I samples were exposed to CO, which is reflected in the subscript CO.

**Table 2:** Summary of the samples investigated and their preparation conditions

Sample	Irradiation	Air exposure	Gas exposure
	time (h)	time (min)	
I1	0	0	$\hbox {N}_{2}$
A1$_0$	2	0	$\hbox {N}_{2}$
A1$_1$	2	1	$\hbox {N}_{2}$
A1$_{30}$	2	30	$\hbox {N}_{2}$
I2	0	0	$\hbox {N}_{2}$
A2	10	0	$\hbox {N}_{2}$
I2$_\textrm{CO}$	0	0	CO
A2$_\textrm{CO}$	10	0	CO

### Whole-cell ESR spectra

**Fig. 1:**
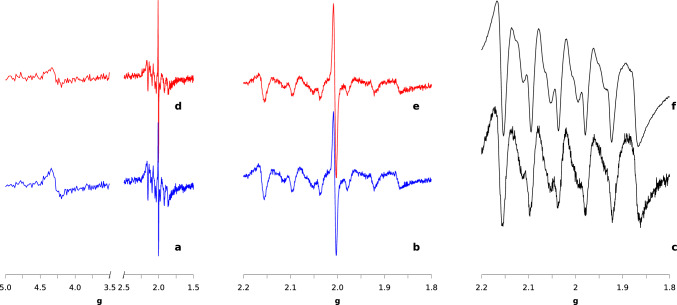
ESR spectra of A2 (**a**, **b**, blue) and I2 (**d**, **e**, red) at a microwave power of 20 $\upmu$W and at $T=$20 K. Panels **b**, **e** display expansions of the spectra shown in a and d, respectively. Panels **c**, **f** show spectrum of $\hbox {Mn}^{2+}$ aquaion and MnBSA, respectively, at the same experimental conditions

In Fig 1 we compare the spectra recorded at the microwave power of 20 $\upmu$W for A2 (blue, bottom) and I2 (red, top) samples at $T=$20 K. Panels c and f display the spectra for MnBSA and $\hbox {Mn}^{2+}$ aquaion, respectively, recorded in the same conditions.

Three intense sets of signals can be observed in both cell conditions. The sextet pattern centered at $g\sim$2.0 and characterized by a hyperfine splitting constant of about 90 G is due to $\hbox {Mn}^{2+}$ ions in octahedral environment (*Mn2* signal, hereafter). This background signal has been observed in many whole-cell experiments [14]. Our *Mn2* pattern is almost identical to that exhibited by the $\hbox {Mn}^{2+}$ aquaion (Fig. 1c) and by MnBSA (Fig. 1f) complexes in the same conditions. Noticeably, the spectrum of $\hbox {Mn}^{2+}$ species at low temperature is almost insensitive to the environment and the two displayed spectra are almost identical to that of the well-studied Mn-concanavalin A (jackbean) complex [27]. Mn is present in the culture medium because cells require it to build many protein assemblies, including photosystem II, the primary sun-light antenna, to sustain normal growth under sun-light. It is interesting to notice that Mn in oxidation state II is visible both after growth in the darkness (Fig. 1d, e) and after weak illumination (Fig. 1a, b). The left-most signal at $g=$4.3 can be assigned to high-spin $\hbox {Fe}^{3+}$ ions that are always visible also in purified FeS proteins [1, 14]. The oxidized iron ions are in rhombic environment, likely an octahedral coordination possibly slightly distorted. This signal will be identified as *Fe3hs*, hereafter. A third signal, centered at $g\sim$2.002$\div$2.005 and largely overlapped to the *Mn2* multiplet, is due to a radical species that is invariably observed even in samples of purified FeS proteins [1, 14]. In photosynthetic cells there are many possible radicals, like quinone pools, flavins, and other intermediates in enzyme reactions, including photosystems. However, most of these radicals are transient species, while those more stable and with a longer life-time are characterized by extended delocalized electron structures, thus producing anisotropic *g* patterns. The radical species that is particularly stable after its formation under light irradiation is the tyrosyl radical (Y$_\textrm{D}$) of the D2 peptide in photosystem II [22]. This isotropic signal will be referred to as *rad* signal, hereafter.

A first-look comparison of the three signals exhibited by I2 and A2 reveals that the difference between the two states resides only in the relative intensities of *Mn2*, *Fe3hs*, and *rad* signals, meaning that the major effect of activation is a different distribution of these species. This point will be analyzed in more detail in Sect. 3.2.

**Fig. 2:**
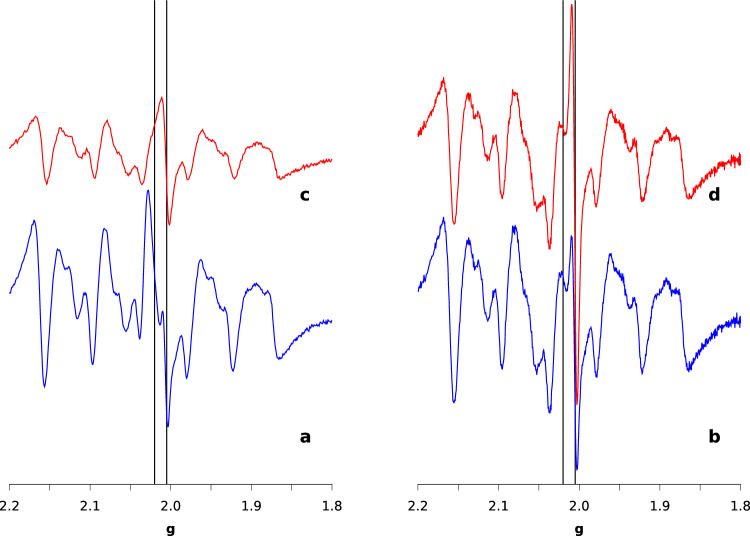
ESR spectra of H2-active samples A1 (**a**, blue), A2 (**b**, blue), and H2-inactive ones I1 (**c**, red), I2 (**d**, red) at a microwave power of 1 mW and at $T=20$ K. Black vertical lines indicates the $g=2.02$ and $g=2.005$ values

We noticed a significant effect of light irradiation time on the ESR spectra. Figure 2 compares ESR spectra in the 1.8$-$2.2 *g* region for I1, A1, I2, and A2 samples.

Firstly, we notice that I1 sample (Fig. 2c) displays a lower *rad* signal compared to I2 (panel d). Secondly, besides the three signals observed in I2 and A2, another signal is visible at $g=$ 2.02 in the samples from preparation 1 (I1 and A1, panels a and c, respectively). This signal is more pronounced in the H2-active sample (A1) compared to their H2-inactive counterpart (I1) and it was tentatively assigned to an FeS cluster on the basis of the *g* value [1, 10, 14]. The signal will be referred to as *FeS* signal, hereafter. The data show that after 10 h of irradiation time the *FeS* signal disappears whereas the *rad* signal increases in intensity. Therefore, the spectral changes in the A1 sample are representative of a transient state that after longer irradiation time (10 h with respect to 2 h) becomes that of A2 and almost identical to that of I2.

In the following we studied temperature dependence and saturation behavior of the signals in the 1.8$-$2.2 *g* range of A1 sample in order to further characterize the state of the addressed paramagnetic centers.

**Fig. 3:**
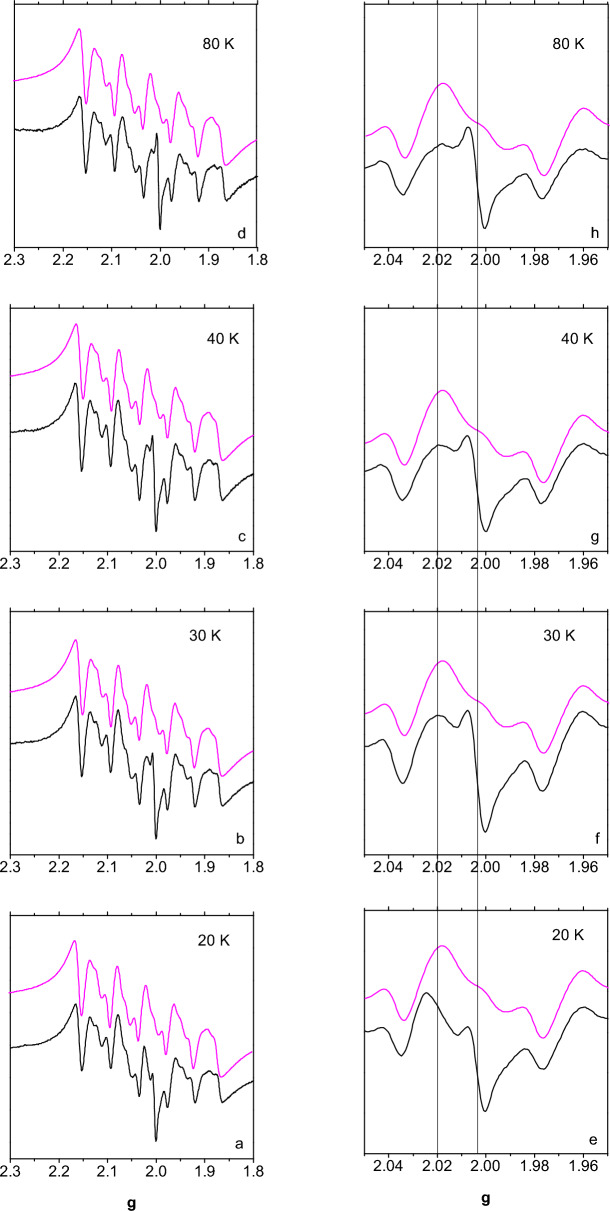
ESR spectra of sample A1 (black line) at a microwave power of 1 mW at the indicated temperatures (**a**–**d**). The spectra are compared with the MnBSA spectrum (purple line) at 20 K. For each spectrum, an expansion is provided on the right-hand side column (**e**–**h**). The vertical lines mark the *g* values of 2.002 and 2.020

The temperature dependence of the $g\sim$2 region is shown in Fig. 3 for sample A1 in the *T* range 20–80 K.

In order to distinguish the signals at $g=2.002$ and $g=2.02$ from that due to $\hbox {Mn}^{2+}$ more clearly, the spectra are compared to that of MnBSA at 20 K. One can notice that the *rad* signal at $g=2.002$ persists up to 80 K, suggesting that it is unlikely due to magnetic states of either $\hbox {Fe}_{4}\hbox {S}_{4}$ or $\hbox {Fe}_{2}\hbox {S}_{2}$ clusters, which usually produce signals that broaden beyond detection above $T=40$ K. Similarly, the *Mn2* pattern does not significantly broaden upon increasing the temperature. In the region around $g=2.02$, the signal observed at $T=20$ K looks different from that recorded at $T=30$ K and from that of *Mn2* in the same spectral region. The shape observed at 30 K persists up to 80 K. This suggests that the *FeS* signal is detectable only at $T=20$ K, consistently with in vitro studies of isolated FeS proteins [1].

The saturation behavior of *Mn2* and *Fe3hs* signals is analyzed plotting $I/\sqrt{P}$ as a function of *P* in a log-log scale, where *P* is the microwave power and *I* is the intensity of each assigned signal.

**Fig. 4:**
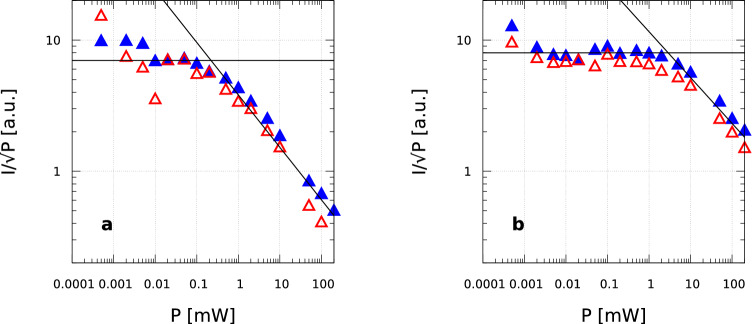
Comparison of the saturation curves for H2-active (A1, blue) and H2-inactive (I1, red) samples of *Mn2* (panel **a**), *Fe3hs* (panel **b**). The intensity, *I*, was measured as the height of the most intense (left-most) peak of the *Mn2* and *Fe3hs* signals in the derivative spectra

The saturation curves are shown in Fig. 4. No significant differences were observed between the H2-active (blue) and H2-inactive states (red) for the two signals. This indicates that the relaxation properties of the $\hbox {Mn}^{2+}$ and high-spin $\hbox {Fe}^{3+}$ species, and therefore their local environments, are not affected by the cellular state. The power of half-saturation, $P_{\small 1/2}$, was derived from the plot, as the intercept of the two straight lines describing the experimental data at low and high microwave powers [4]. For *Mn2* and *Fe3hs*, we found $P_{\small 1/2}=$ 0.2 mW and 2 mW, respectively.

**Fig. 5:**
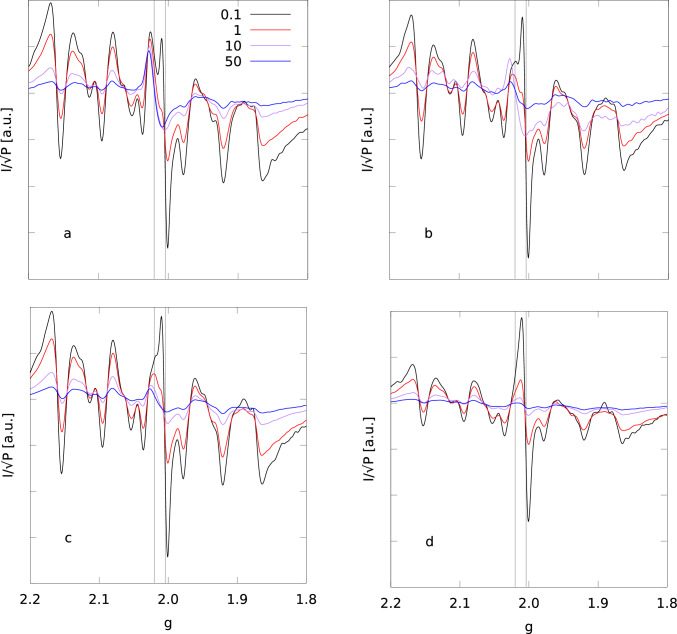
Comparison of ESR spectra obtained at different microwave powers for H2-active (sample A1$_0$) (**a**), and after 1 (A1$_1$, **b**), and 30 (A1$_{30}$, **c**) minutes of air exposure; The spectra of H2-inactive sample (I1) are shown in panel (**d**). Microwave power is 0.1 mW (black), 1 mW (red), 10 mW (purple), and 50 mW (blue). Temperature is 20 K

The same analysis can not be performed for the *FeS* and *rad* signals because of the large signal overlap. However, the simple inspection of the spectra recorded at different power in I1 and A1 samples is very indicative of possible crucial differences between cellular states. In Fig. 5 we display the spectra for I1, and H2-active after 0 (A1$_0$), 1 (A1$_1$), and 30 (A1$_{30}$) minutes of air exposure (see Table 2), selecting the field range corresponding to 1.8$-$2.2 in *g*. In this way we emphasize the different behavior of *rad* and *FeS* signals.

The intensity of the *FeS* signal at $g=$ 2.02 persists up to $P=$ 50 mW in all the H2-active samples (Fig. 5a), whereas it is strongly attenuated at the high powers in I1 (Fig 5d). The attenuation of *FeS* signal in A1$_1$ and A1$_{30}$ is intermediate between A1$_0$ and I1.

**Fig. 6:**
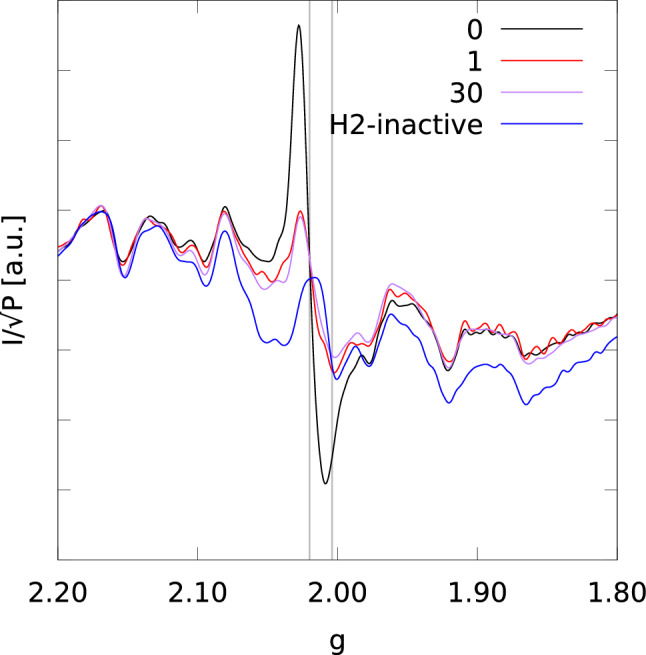
Comparison between spectra of different samples (I1, A1$_0$, A1$_1$, and A1$_{30}$) at microwave power 50 mW. A1$_0$ (black curve); A1$_1$ (red); A1$_{30}$ (purple); I1 (blue)

This is clearly shown in Fig. 6, where spectra recorded at $P=$ 50 mW are shown for all I1 and A1 samples. In summary, these data show that the transient signal at $g=$ 2.02 is due to an oxygen sensitive species that is characterized by a fast magnetic relaxation.

According to the existing literature about FeS clusters [26], in the following we tentatively attribute the *FeS* signal to a specific type of FeS cluster. The most abundant FeS multinuclear clusters are $\hbox {Fe}_{4}\hbox {S}_{4}$ and $\hbox {Fe}_{2}\hbox {S}_{2}$ in oxidation states dependent on the cellular state and compartment. In resting state of most of the iron-sulfur proteins, the former cluster is in the $\hbox {Fe}_{4}\hbox {S}_{4}{^{2+}}$ state that is diamagnetic [35] and therefore silent in ESR. The latter cluster is one half of the former, $\hbox {Fe}_{2}\hbox {S}_{2}{^{+}}$, and it displays a highly rhombic pattern spreading *g* within 2.04$-$1.90 region. We do not observe an evident anisotropic signal in our spectra in this region.

Other commonly observed FeS clusters are $\hbox {Fe}_{4}\hbox {S}_{4}{^{3+}}$, $\hbox {Fe}_{4}\hbox {S}_{4}{^{+}}$, and $\hbox {Fe}_{3}\hbox {S}_{4}{^{+}}$ [1, 10, 14, 26]. The first cluster is characterized by an axial pattern of *g*, but it tends to rapidly expel one Fe atom. The second cluster has been investigated in ferredoxin [11] and it is also characterized by a rhombic *g* pattern [26]. Only the third cluster displays an almost isotropic signal and it is therefore compatible with our data.

The contribution to the signal from transient species related to the assembly of the specific [FeFe] hydrogenase H-cluster primarily originates from species involved in $\hbox {Fe}_{4}\hbox {S}_{4}$ biogenesis, species that are abundant in algae cells. In microorganisms, iron-sulfur clusters, formed by Fe, sulfide ions and Cys sidechains have larger chance to form compared to heme and other octahedral mononuclear Fe binding sites (like the hydrogenase H-cluster). Heme and H-cluster sites require specific ligands, rather than abundant sulfur and Cys ligands. Therefore, a deeper analysis can be done by using published data addressing the mechanism of FeS cluster oxidation. The mechanism of oxidation has been studied in vitro by using fumarate and nitrate reduction regulators in *E. coli* [7, 8, 23]. These studies identified $\hbox {Fe}_{3}\hbox {S}_{4}{^{+}}$ as the most persistent intermediate in the oxidation of $\hbox {Fe}_{4}\hbox {S}_{4}{^{2+}}$ by dioxygen. The species is the product of the first fast step in oxidation, because $\hbox {Fe}_{4}\hbox {S}_{4}{^{3+}}$ is so unstable that rapidly releases $\hbox {Fe}^{2+}$ to the environment:

3 $$\begin{aligned} {[}\hbox {Fe}_{4}\hbox {S}_{4}{]}{^{2+}} + \hbox {O}_{2} \longrightarrow {[}\hbox {Fe}_{3}\hbox {S}_{4}{]}^{+} + \hbox {Fe}^{2+} + \hbox {O}_{2}{^{\cdot -}}, \end{aligned}$$

where superoxide anion is usually rapidly degraded in the chloroplast by Mn- and Fe-SOD, both containing the reduced form of the respective Mn and Fe ions. The second step involving the release of Fe from $\hbox {Fe}_{3}\hbox {S}_{4}{^{+}}$ to produce the $\hbox {Fe}_{2}\hbox {S}_{2}$ cluster is slow, thus allowing a transient detection of the $\hbox {Fe}_{3}\hbox {S}_{4}{^{+}}$ intermediate.

4 $$\begin{aligned} {[}\hbox {Fe}_{3}\hbox {S}_{4}{]}{^{+}}\longrightarrow {[}\hbox {Fe}_{2}\hbox {S}_{2}{]}^{2+} + \hbox {Fe}^{3+} + 2 \hbox {S}_{2}{^{2-}}. \end{aligned}$$

The spectral features of $\hbox {Fe}_{3}\hbox {S}_{4}{^{+}}$ have been reported by using model peptides (maquettes) stabilizing it [15]. The model compounds allowed to assign an almost isotropic signal at $g=$ 2.01 to $\hbox {Fe}_{3}\hbox {S}_{4}{^{+}}$. This signal shares most of its features with the one observed by us in whole cells as the *FeS* signal. The decrease of *FeS* signal from H2-active state to H2-inactive state observed by us can be interpreted as the exhaustion of the oxidation intermediate ($\hbox {Fe}_{3}\hbox {S}_{4}{^{+}}$) occurring when dioxygen amount increases.

Therefore, we attribute *FeS* signal to the species $\hbox {Fe}_{3}\hbox {S}_{4}{^{+}}$. So far, by ESR we found the maximal intensity of this FeS species in the conditions where the production of $\hbox {H}_{2}$ is at the beginning, that is after 2 h of low-intensity light irradiation. Although this state (A1) is H2-active, many processes are expected to occur in the cells before achieving state A2 and the maximal $\hbox {H}_{2}$ production occurs.

The exposure to CO gas of cells before injection into ESR tubes (samples I2$_\textrm{CO}$ and A2$_\textrm{CO}$) shows that there is no difference in ESR spectra (data not shown). The spectra are identical to those of cells exposed to $\hbox {N}_{2}$ only (Figs. 1, 2b, d). We could not collect spectra after short (2 h) irradiation time, but the passive CO penetration into cells is likely slower than in cell cultures and these experiments will be repeated with a proper equipment under controlled pressure and time conditions on in vivo cell cultures.

In summary, we speculate that the transient species addressed after 2 h irradiation represents a buffer of immature FeS clusters that can be used in two pathways: (i) assembling/degradation of functional $\hbox {Fe}_{4}\hbox {S}_{4}{^{2+}}$ clusters, mostly used by enzymes in reducing conditions; (ii) assembling/degradation of H-cluster in mature hydrogenase. The *FeS* signal we observe in this work for samples after a short time of light irradiation is a sort of marker of a FeS cluster round-about formed after heterotrophical growth in the dark of unicellular algae.

### Cellular concentration of ESR active species in different states

Total Fe and Mn content in all the analyzed samples, as determined by ICP-OES, is reported in Table 3.

**Table 3:** Comparison between I2 (H2-inactive) and A2 (H2-active) states in terms of iron and manganese total content ($\upmu$g) and number of Fe and Mn atoms per cell

Sample	Cell density (cell/l)	Fe total ($\upmu$g)	Fe atoms per cell (atoms/cell)	Mn total ($\upmu$g)	Mn atoms per cell (atoms/cell)
I2	6.4 ± 0.3 10$^{11}$	1.0 ± 0.1	2.8 ± 0.4 10$^7$	0.22 ± 0.02	6.3 ± 0.9 10$^6$
A2	5.3 ± 0.1 10$^{11}$	1.6 ± 0.2	5.4 ± 0.7 10$^7$	0.27 ± 0.01	9.3 ± 0.4 10$^6$

The cell density is also reported. Total Fe and Mn content is obtained by ICP-OES

Ni, Co and Cu content was lower than detection limit (0.05, 0.05 and 0.1 $\upmu$g, respectively).

The number of Mn and Fe atoms per cell has significantly increased during phototrophy, going from I2 to A2. The number of Mn atoms per cell in I2 corresponds to a concentration of Mn of 160 $\upmu$M, assuming that the diameter of a cell is equal to 5 $\upmu$m. As expected, this concentration largely exceeds that of the growth medium, equal to 31 $\upmu$M. The order of magnitude of Mn atoms per cell found by us is similar to that measured with microanalytical techniques in similar growth conditions for *Cvu* cells [31]: $\sim$10$^8$ Mn atoms per cell.

Our data show that during photoproduction of $\hbox {H}_{2}$ (going from I2 to A2) the cell density decreases, but the number of Mn and Fe atoms inside each cell increases, with a significantly higher internalization of Fe than Mn.

Then the ESR spectra can be used to distribute the total amount of ions within the oxidation states. We used the ESR spectra under non-saturating conditions to estimate the relative amount, *R* (as defined in Eq. 2), of $\hbox {Mn}^{2+}$ ions and high-spin $\hbox {Fe}^{3+}$ ions per cell in the H2-inactive state relative to the H2-active state. The values of *R* obtained at the two irradiation times are provided in Table 4.

**Table 4:** Manganese and high-spin oxidized relative content (*R* in Eq. 2) for H2-inactive/H2-active state in samples I1, A1, I2, and A2 (see Table 2)

	$\hbox {Mn}^{2+}$	*hs*-$\hbox {Fe}^{3+}$
I1/A1	0.63	2.0
I2/A2	0.71	0.51

The number of Mn(II) ions per cell in H2-active cells is 1.4$\div$1.6 times that in H2-inactive cells at both light irradiation times. Since the ratio of total Mn and Mn(II) ions in the two states is about the same (0.6$\div$0.7), we argue that the amount of Mn that is internalized is equally partitioned between Mn(II) and oxidized states during phototrophy. The ratio between reduced (II) and oxidized Mn is kept constant, no matter of irradiation time.

The cells in the H2-inactive state have the photosystem ready to work, as shown by the photosynthetic activity when they are put under the light and $\hbox {H}_{2}$ production starts. Mn ions of the oxygen evolving center, part of photosystem II, are in oxidation states higher than II. These oxidized states are observed in ESR at conditions different from ours [25]. Mn(II) is the cofactor essential to enzymes that are involved in scavenging various reactive oxygen species (ROS). Among these enzymes, the most representative ones are dinuclear Mn catalases and Mn-superoxide dismutase (Mn-SOD) [9]. Our approximate estimate of Mn(II) pool in different cellular states of *Cvu* shows that the two Mn functions (oxygen production through the oxygen evolving center and ROS scavenging) are increased in parallel during phototrophy.

Conversely, the H2-active state displays 1/2 of the amount of high-spin $\hbox {Fe}^{3+}$ displayed by H2-inactive cells, when the $\hbox {H}_{2}$ production is in the early stage (sample A1). The ratio of high-spin $\hbox {Fe}^{3+}$ is inverted in the later stage of $\hbox {H}_{2}$ production (sample A2), showing that unbalance of oxidized iron due to oxidative stress is changed: iron is incorporated into cells along with light irradiation time in the H2-active state (Table 3); the number of high-spin oxidized single-iron species per cell increases (Table 4); the organic radical ($g\sim$2) increases (Fig. 2, panel e). In conclusion, the increase of relative high-spin oxidized iron is a marker of late oxidation by the increasing amount of dioxygen.

The affinity of both $\hbox {Mn}^{2+}$ and $\hbox {Fe}^{3+}$ for abundant metal ions’ transporters present in cell cytoplasm, like metallothionein, prevents the observation of free ions in whole cells. However, the *Fe3hs* signal is characteristic of single-ion sites like that evident in water, where Fe(III) is in a distorted octahedral coordination. Therefore, high-spin oxidized iron is likely in mononuclear complexes rather than in multinuclear clusters, the latter stabilizing reduced iron by protection with sulfur anions.

The behavior of the ratio of *Fe3hs* species can be interpreted according to the proposed oxidation pathway of the most stable reduced FeS clusters, that was described above (Eqs. 3-4) and that is summarized in Ref. [8]. The oxidation of reduced FeS clusters occurring when the production of $\hbox {O}_{2}$ increases (10 h of irradiation) creates a pool of oxidized iron ions not embedded in Fe multinuclear clusters. At the early stages of oxidation, when $\hbox {O}_{2}$ is at lower concentration, the transient $\hbox {Fe}_{3}\hbox {S}_{4}{^{+}}$ cluster can be visible and oxidized iron is still kept under sulfur bonds. The formation of persulfide bonds on Cys residues occurs along with the expulsion of $\hbox {Fe}^{3+}$ cations and this step slowly produces the organic radical likely on the glycyl $\alpha$ carbon [24]. The localization of unpaired electrons on glycine can explain the observation of the *rad* signal in samples containing isolated iron-sulfur proteins [1].

## Conclusion

For the first time, whole-cells of a promising $\hbox {H}_{2}$ producing *Chlorella vulgaris* strain (g120) were observed by ESR spectroscopy. Observations were done: i) before hydrogen production starts (H2-inactive state, samples I1-I2); ii) when hydrogen production begins (H2-active state achieved after 2 h of low-intensity light irradiation, sample A1); iii) when hydrogen production is full (H2-active state after 10 h of low-intensity light irradiation, sample A2). The spectra are remarkably simple in both states (I and A), containing useful information about 4 spin systems: reduced Mn; oxidized high-spin iron; an ubiquitous radical; one spin system with high relaxation rate identified as a peculiar, likely immature, FeS cluster visible only at early hydrogen production and when samples are not exposed to air.

By modifying experimental conditions and comparing results to the available literature data about isolated FeS systems, we could assign the last signal to $\hbox {Fe}_{3}\hbox {S}_{4}{^{+}}$. Together with measured Mn and Fe internalization and changes of the amount of reduced Mn and oxidized high-spin iron upon changes in cellular state, we extend a proposed mechanism of iron trafficking via FeS clusters upon changes of oxidoreductive conditions, including dioxygen sensing. In this mechanism, $\hbox {Fe}_{3}\hbox {S}_{4}{^{+}}$ species acts as a transient buffer of pre-formed FeS clusters that allow a fast achievement of high levels of $\hbox {Fe}_{4}\hbox {S}_{4}{^{2+}}$ clusters that are functional in many biological processes, including the maturation and activity of [FeFe] hydrogenase.

The experiments and their analysis can be performed with no critical requirements, like glove-boxes and strictly anaerobic conditions required for oxygen-sensitive isolated enzymes. Working under $\hbox {N}_{2}$ stream during cell preparation is sufficient to avoid uncontrolled changes of cellular state. On the other hand, results strongly depend on the cell preparation in the H2-active state, particularly on the light irradiation time. Therefore, the reported data will be the basis for an extended analysis of whole-cells measurements, including other spectroscopical and microscopy techniques, and changes in cellular environment.

## Supplementary Information

Below is the link to the electronic supplementary material.

Supplementary file 1 (pdf 85 KB)

## Data Availability

All data can be requested to corresponding author.

## Abbreviations

ESR: Electron spin resonance
CW: Continuous wave
*Cr*: *Chlamydomonas reinhardtii*
*Cvux*: *Chlorella vulgaris*
BSA: Bovine serum albumin
Mn2: Mn(II) signal
Fe3hs: High-spin Fe(III) signal
FeS: Iron–sulfur signal
rad: Radical signal
H-cluster: Hydrogenase active site
ROS: Reactive oxygen species
