# Supplementary material for: Electron spin resonance in microalgae whole-cells to monitor hydrogen production
Source: J Biol Inorg Chem. 2025 Mar 24;30(3):229–40. doi: 10.1007/s00775-025-02113-0 (PMC11965266; doi:10.1007/s00775-025-02113-0)
Supplement: Supplementary file 1 — (pdf 85 KB) [file 775_2025_2113_MOESM1_ESM.pdf]

Electron Spin Resonance in Microalgae Whole-cells to Monitor Hydrogen Production

Silvia Pizzanelli[1], Emanuela Pitzalis[1], Simone Botticelli[2,3], Fabrizio Machetti[5], Cecilia Faraloni[4], Giovanni La Penna[5,3*]

[1]Institute of chemistry of organometallic compounds, National research council, via Moruzzi 1, Pisa, 56124, Italy.
[2]Department of physics, University of Roma Tor Vergata, via della Ricerca Scientifica 1, Roma, 00133, Italy.
[3]Section of Roma Tor Vergata, National institute for nuclear physics, via della Ricerca Scientifica 1, Roma, 00133, Italy.
[4]Institute of bioeconomy, National research council, via Madonna del Piano 10, Sesto Fiorentino (Firenze), 50019, Italy.
[5]Institute of chemistry of organometallic compounds, National research council, via Madonna del Piano 10, Sesto Fiorentino (Firenze), 50019, Italy.

*Corresponding author(s). E-mail(s): giovanni.lapenna@cnr.it;
Contributing authors: silvia.pizzanelli@cnr.it; emanuela.pitzalis@cnr.it; simone.botticelli@roma2.infn.it; fabrizio.machetti@cnr.it; cecilia.faraloni@cnr.it;

Supplementary Information

1 ESR spectra elaboration

Spectra acquired in Bruker™ format were converted to text files by using MatLab [2] and the Easyspin package (version 6.0.6) [4]. The text files were loaded with R programs [3].

Each of the $I(B)$ functions were subtracted of the cavity signal acquired at the beginning of the same day of measurements and at the same microwave power. The resulting functions were corrected for an evident decrease in base-line. When the spectra were acquired at a window of 5000 G the first and last 75 points were used, while 150 points for each end were used for the 1000 G window. These points define the constraints of the base-line. The missing points of the base-line were interpolated using cubic spline in R and the Forsythe, Malcolm and Moler method (fmm) [1].

In Fig. S1 the elaboration is shown for one of the replicate I3 sample. See Methods for details.

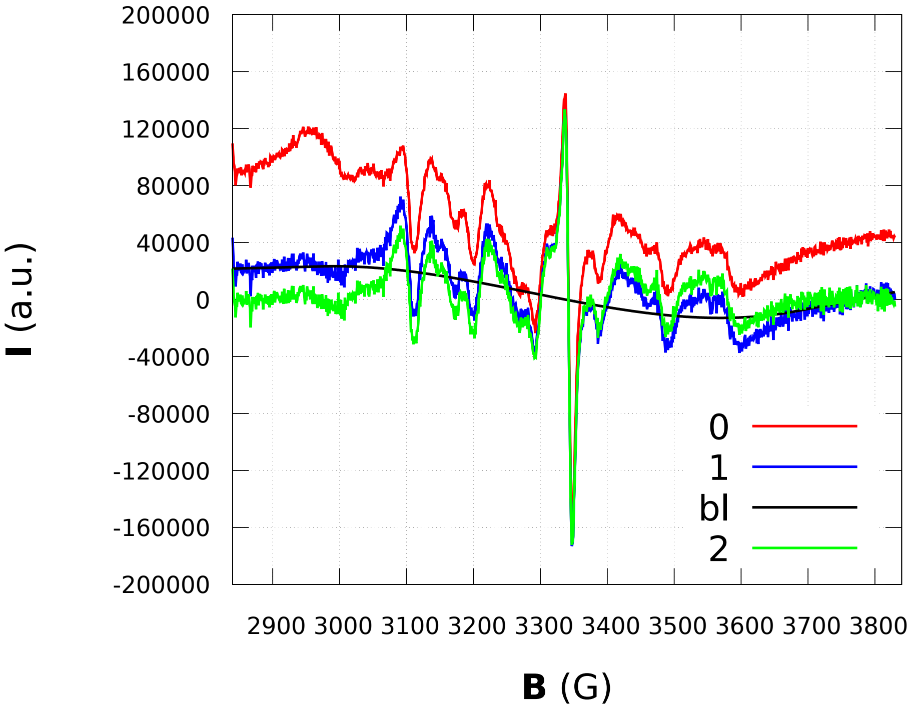

Figure S1: Spectrum elaboration of one replicate I3 sample (power is 1 mW, field window is 1000 G). Red - raw data (0); blue - result of cavity subtraction (1); black - base-line obtained by the interpolating cubic spline constrained to the 150 end points on each side of the blue curve (bl); green - corrected spectrum (2).

Once each of the spectra was corrected, they were averaged over replicate experiments. As for the given example (I3), the average result over the 3 replicates is presented in Fig. 2d in the manuscript.

Finally, the x-axis (B) of each spectrum was converted to g values using:

$$g = \frac{h\nu_0}{\mu_B B} \ , \tag{1}$$

where μ_B is the Bohr magneton, h is the Planck constant, the magnetic field B is in T, and ν_0 is the average, over replicates, of the frequency determined by the static magnetic field (MWFQ parameter of the Bruker DSC file).

References

[1] Forsythe GE, Malcolm MA, Moler CB (1977) Computer Methods for Mathematical Computations, vol XI. Prentice Hall, Inc., Englewood Cliffs, New Jersey, USA

[2] MATLAB (2022) R2022a. The MathWorks Inc., Natick, Massachusetts, https://www.mathworks.com

[3] R Core Team (2021) R: A Language and Environment for Statistical Computing. R Foundation for Statistical Computing, Vienna, Austria, URL https://www.R-project.org/

[4] Stoll S, Schweiger A (2006) Easyspin, a comprehensive software package for spectral simulation and analysis in epr. J Magn Res 178(1):42–55. https://doi.org/10.1016/j.jmr.2005.08.013
